# Implant insertion torque value in immediate loading: A retrospective study

**DOI:** 10.4317/medoral.22845

**Published:** 2019-04-24

**Authors:** Roberto Del Giudice, Adriano Piattelli, Nicola-Maria Grande, Enrico Cataneo, Antonio Crispino, Morena Petrini

**Affiliations:** 1 DDS, Private Practice, Catanzaro, Italy; 2 Full professor of Oral Pathology, Department of Medical, Oral and Biotechnological Sciences, University of Chieti-Pescara, Chieti, Italy; 3 DDS, Ph.D, Professor of Endodontics, Catholic University of Sacred Hearth, Rome, Italy; 4 DDS, Adjunct Professor of Prosthodontics, Department of Odontostomatology, “Magna Graecia University” of Catanzaro, Italy; 5 DDS, Ph.D, Private Practice, Catanzaro, Italy; 6 DDS, Ph.D, Oral Surgery Specialist, Research Assistant, Department of Medical, Oral and Biotechnological Sciences, University of Chieti-Pescara, Chieti, Italy

## Abstract

**Background:**

The aim of this study is to verify if the Insertion Torque Value (ITV) of 32 Ncm for immediate loading protocol (ILP), as indicated by literature, is still, with the advance in implant research, a real significant cut-off for long-term implant survival.

**Material and Methods:**

In this retrospective study, data from 224 patients that during three years of clinical practice, were submitted to the insertion of 322 implants with immediate loading protocol, have been recorded, pooled and analyzed. Data were organized based on Insertion Torque Value (ITV): > 32 Ncm (CG) and < 32 Ncm (LTG) and two different groups of equal sample size, 161 implants each, were distinguished. Crestal bone reabsorption, and the implant failure rate were evaluated after 2-years of follow-up.

**Results:**

The bone reabsorption in LTG (0.49 ± 0.11 mm) was significantly greater than CG (0.22 ± 0.04 mm), *p*<0.001. However, the survival rate after 2-years of follow-up was quite high and similar for both groups: 96.89% for LTG and 97.52% for CG and no statistically significant differences have been found among the two groups for the implant failure rate (*p*=0.455). The Odds Ratio (OR) of implant failure was of 1.258 (95% CI 0.332, 4.772), but results were not statistical significant, *p*=0.740.

**Conclusions:**

The present study showed that although implants with ITV> 32 Ncm are still characterized by a lower crestal bone resorption, there are no statistically significant differences among the two groups for what concerning the failure rate during the 2 years of follow-up and OR. These results permit us to suppose that the cut-off of ITV >32 Ncm for immediate loading implants, could be reduced to inferior values. However further studies are necessary to indicate precise clinical guidelines.

** Key words:**Immediate loading, insertion torque, primary stability, dental implants, implant survival.

## Introduction

A good primary stability and a direct interface between the implant surface and the alveolar bone during the healing process is fundamental for osteointegration (1).

Traditionally, it was assumed that to achieve osseointegration, implants needed to be submerged under the mucosa and left without any loads for a period of 3–6 months (2). Then, patients needed to be submitted to a second stage surgery, and before to be restored with the final restorations, they had to undergo different schedules, with a great sacrifice of time, aesthetics and function (3).

With the advent of the immediate loading protocol (ILP), it is no longer necessary to wait this time and inconveniences; the implant can be loaded immediately after the surgical phase or within 48h (4,5). Compared to conventional delayed loading, ILP is convenient and comfortable for patients because it permits to achieve functional and aesthetical results, immediately after the implant insertion. The recent International Team for Implantology Consensus Conference showed that ILP for single-tooth restorations seems to positively influence patients oral health-related quality of life respect conventional protocols. (6).

However, Esposito and coworkers included ILP as one factor in the increased rate of complications in implant dentistry (7). Moreover, a recent review published in 2015 has shown that ILP imposed a significantly higher risk of implant failure than did conventional loading (risk ratio = 2.09, 95% confidence interval [CI] [1.18, 3.69], *P* = 0.01) (8).

In order to use ILP, a high degree of implant primary stability measured by ITV must be verified intraoperative, as shown by Testori and coworkers (9).

ITV is a biometric parameter that can be influenced by several conditions, such as the size of the recipient site, the morphology and quality of the bone, the macro and micro design of implants, and the surgical technique (10,11). Under-preparation of the recipient site seems to be the most common technique to increase the pressure to the bone while inserting implants. This may result in higher ITV when compared with conventional implant bed preparation (12,13) .

Many authors refer to their stability criteria for ILP an ITV greater than 30 Ncm (14,15); an important variable is the type of final restoration that the implant will support: Calandriello *et al.* indicated a minimum ITV of 60 Ncm for single teeth, 45 Ncm for implants supporting partial-arch restorations, and 32 Ncm for implants supporting full-arch restorations (16).

A recent review of Huynh-Ba *et al.* highlighted that most of the literature on ILP is based on implants with ITV > 30 Ncm (6); in the study of Felice *et al.* published in 2011, implants with torque values inferior to 35 Ncm, were left to heal for 4 months before proceeding with the loading (17).

However, the constant progress on the osteology research, biomaterials and implant design and surface developments are continually challenging the initial treatment guidelines.

The purpose of this work is to understand if the cut-off of ITV>32 Ncm for immediate loading protocol, as indicated from literature (15,18-21) is still, with the advent of new implant surfaces and designs, a real significant criteria or not.

## Material and Methods

In this retrospective study, data from medical records of 224 Caucasian patients, that in the previous years were subjected to the dental implant insertion with immediate loading protocol (ILP), were selected, pooled and analyzed.

A different Arabic number (code) was assigned to each implant, in order to distinguish from each other. Data were organized in two groups, basing on the Insertion Torque Value (ITV): conventional group, CG, with ITV >32 Ncm, and lower torque group, LTG, with ITV <32 Ncm.

-Inclusion and Exclusion Criteria 

Inclusion criteria were: dental implants placed in the healed bone or in fresh extraction sockets, with ITV recorded and loaded with ILP with at least two years of follow-up.

Other inclusion criteria were: bone volume enough to insert implants of suitable diameter without interventions of bone regeneration, the absence of disease that could affect bone healing, good general health, controlled oral hygiene, age between 18 and 75 years.

Exclusion criteria were: severe parafunctions, severe intermaxillary discrepancy, drugs or alcohol addiction, poor oral hygiene, gestation or lactation, previous radiation therapy, periodontal disease untreated or unresolved.

-Surgical and Prosthetic Procedures

Diagnostic models, endoral and panoramic radiographs, and computed tomography have been examined, before surgery, for each case.

Before the surgery, all patients were informed about the procedures, and then they signed the informed consent; then they assumed a prophylactic antibiotic therapy (2g of amoxicillin or 1g of clarithromycin – if allergic to penicillin) and rinsed for 1 minute with chlorhexidine mouthwash 0.2%.

Local Anesthesia was performed with mepivacaine with adrenaline 1:100,000. Implants of various diameters and lengths (Osseotite; Biomet 3i, Palm Beach Gardens, FL, USA) were positioned according to the manufacturer’s instructions.

The ITV was measured intraoperative, through an implant micromotor (up to 80 Ncm) or the torque wrench (greater than 80 Ncm) and recorded.

The electronic resonance frequency analysis, RFA (Osstell, Integration Diagnostics AB, Göteborg, Sweden), was used to measure the fixture implant stability during the insertion and at the time of the final impression (4 and 6 months after surgery for Misch quality scale (22) D1/D2 and D3/D4, respectively). Immediately after surgery, a provisional restoration was connected to the fixtures; only rehabilitations greater than three elements were loaded with functional occlusion.

A postoperative antibiotic therapy 6 or 8 hours after surgery (1g of amoxicillin or 500 mg of clarithromycin) was prescribed for all patients.

The time of definitive load varied according to the bone quality scale (22); for bone quality D3/D4: approximately after 6 months from surgical time and for bone quality D1/D2: approximately after 4 months from surgical time. All final prostheses were loaded with a functional occlusion.

-Follow-Up

All patients were seen 7 days after surgery. Survival rate was evaluated at 2-years of follow-up in which patients were observed once a month until definitive load, and then, once every 6 months. Criteria used for the survival rate were those proposed by Albrektsonn (23) and were defined as the absence of the following conditions: implant mobility, the persistent and/or irreversible signs and symptoms such as pain, infections, neuropathies radiographic periimplant radiolucency and a vertical bone loss greater than 0.2 mm annually following the first year from implant insertion.

Bone reabsorption was evaluated on digital endoral radiographs, by the use of a digital ruler (a custom occlusal jig permitted to make the examination repeatable in time), that measured the distance from implant’s shoulder to first bone-to-implant contact (BIC). The evaluation of bone reabsorption started since the fixtures were connected with definitive prostheses.

-Statistical analysis

All medical data were recorded in data sheet and then the statistical analysis was performed using SPSS for Windows version 21 (IBM SPSS Inc., Chicago, IL, USA). The mean values ± standard deviation of ITV, average bone reabsorption and survival rate were calculated. The statistical analysis was performed through the Student’s T-Test with two tails for independent variables. The significance threshold was set at 0.05.

The Odds Ratio for implant failure was also calculated through the free online software MedCalc Software (https://www.medcalc.org/calc/odds_ratio.php).

## Results

**Figure 1:**
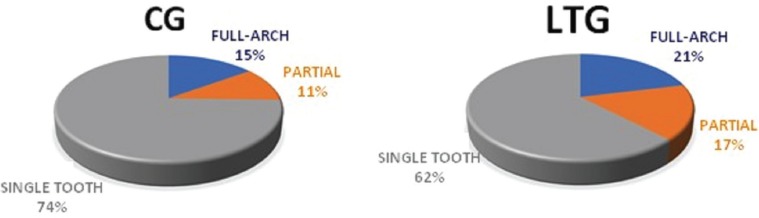
Prosthetic rehabilitations supported at baseline.

The implants analyzed in this study were of variable measures, diameters and lengths ( Table 1), were positioned in different alveolar sites ( Table 2) and supported different types of prosthetic rehabilitation (Fig. 1). The surgical technique was in 72% of cases flapless, while in 28% of cases conventional periosteal flap. The provisional prosthetic restoration was screwed in 66% of cases and cemented to the abutment in the 34%.

**Table 1:**
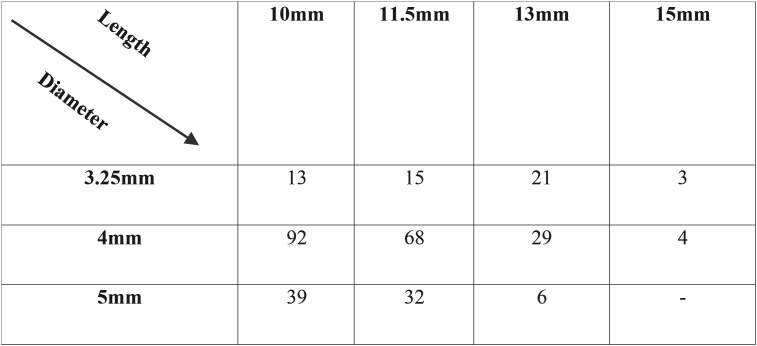
Lengths and Diameters of Implants (number) included in the study.

**Table 2:**
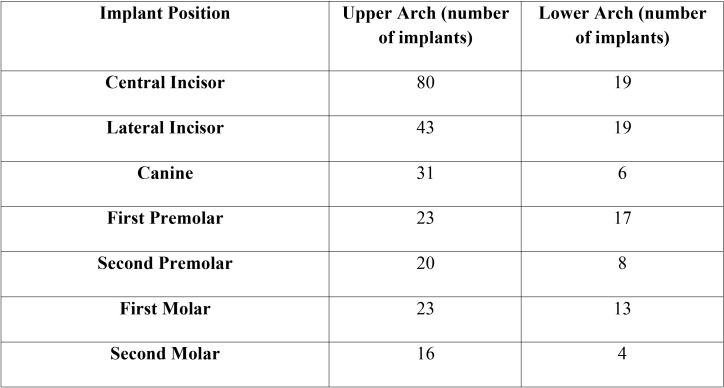
Anatomical Position of Implants (number).

Overall healing was optimal, the swelling was minimal, and patients reported no lingering pain in the 7 days after surgery.

The connection of definitive prosthesis was screwed in 22% of cases and cemented in 78% of cases. Materials used for definitive crowns were metal-ceramic (87%) and integral ceramic (13%).

The survival rate after 2-years of follow-up was of 97.52% for CG and 96.89% for the LTG. The failure rate was low for both groups and there were not statistically significant differences: 3.11% (5/161) and 2.48% (4/161) for LTG and CG, respectively (*p*=0.455).

**Figure 2:**
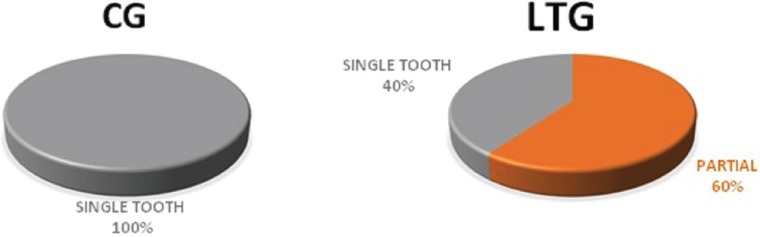
Prosthetic rehabilitations of the failed implants, after 2 years of follow-up.

It is important to highlight that implants that failed in in the LTG group, supported both partial prosthesis (3 implants) than single-tooth restorations (2 implants), ( Table 3, Fig. 2); on the contrary, those that failed in the CG groups, (4 implants) supported all single-tooth restorations ( Table 4, Fig. 2); this result is very important, because there were statistically significant differences for what concerning the prosthetic rehabilitations of the failed implants in the 2 groups, *p*<0.001.

**Table 3:**
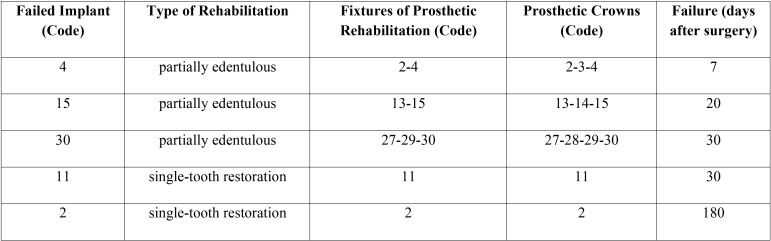
Failed Implants (LTG) and types of rehabilitation in which they are involved (ADA Nomenclature).

**Table 4:**
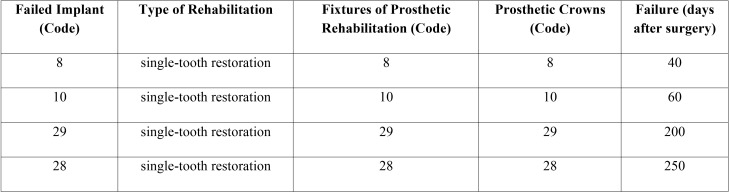
Failed Implants (CG) and types of Rehabilitations in which they are involved (ADA Nomenclature).

Also the average timing of implant failure was very different among the two groups: 137.50+103.40 (CG) and 53.40+71.40 (LTG) days after the insertion, however, results were not statistically significative (*p*=0.170), ( Table 3, Table 4).

The calculated Odds Ratio for implant failure was 1.258 (95% CI 0.332, 4.772), *p*=0.740 for LTG.

The average ITV was 61.30 ± 12.84 Ncm and 24.03 ± 4.07 Ncm, for CG and LTG, respectively.

Average bone reabsorption, calculated from the time of definitive load until the end of 2-years of follow-up was 0.22 ± 0.04 mm and 0.49 ± 0.11 mm for CG and LTG, respectively.

Although the failed implants were not included for the calculation of the average bone reabsorption, the statistical analysis has shown significant differences between test and CG (*p*<0.01).

## Discussion

Both protocols permitted to reach a survival rate after 2 years of follow-up that is similar with the percentage (weighted mean value 97.90% at 24.3 months) shown by Gallucci *et al.* in the recent review about loading protocols of partial rehabilitations (24). LTG results seem to be in agreement with a study of Barewal and coworkers which showed that was possible to reach both long-term success and optimal tissue response, by using a cut-off for ITV of 20 Ncm and 10 Ncm for immediate and early loading, respectively (25).

Contrarily, a study of Ottoni and coworkers showed a severe failure rate: 9 implants on 10 inserted with an ITV of 20 Ncm and immediately loaded, as part of a larger group test, failed (18). Another limitation is that we considered only ITV, but we did not had data about the Resonance frequency analysis (RFA) performed and for this reason we cannot insert this parameter in the statistical analysis. Many studies claimed that lower ITV, comprised between 35 and 15, combined with RFA > 50 ISQ were a significant indicator for ILP or early one (24,26,27).

It is important to highlight that the technological improvement of implant surfaces and the surgical tools and the refinements of the surgical protocols could have positively influenced our results, respect literature of the past 10 years.

The Odds ratio was 1.258 for implant failure but the results were not statistically significant (*p*=0.740). The peri-implant crestal bone reabsorption appears to be lower for CG than implants of the LTG. Statistical analysis shows a significant difference between control and LTGs. This might be explained by the fact that the implants screwed with a greater ITV, CG, were more stable than implants of the LTG and therefore less free to carry out micro-movements in order of microns; hence, around them, it was possible an healing without excessive remodeling of peri-implant bone tissue. This finding is in agreement with an animal study performed by Rea and coworkers that demonstrates how the peri-implant bone reabsorption is greater for implants placed with a lower ITV during the surgical phase(28). Another important fact that emerges from this study is the finding that none of the failed implant supported total rehabilitations. This is in agreement with a recent study carried out by De Bruyn and coworkers which point out that for immediate loading protocol, survival rates are greater for full-arch rehabilitation than partial ones or single tooth restorations (29). Moreover, we must highlight that 3 of the 5 failed implants in the LTG, were placed with an immediate post extractive protocol, and the other 2 were placed in D4 quality bone tissue.

It is important to highlight that the Albrektsson criteria adopted in this study to evaluate the implant failures, considered only survival parameters, not the successful ones (23). This could create some bias, because implants that meet survival criteria, could not meet aesthetic ones.

## Conclusions

The results obtained in this study suggests that the cut-off for ITV of 32 Ncm, for ILP, could be reduced to inferior values making possible the extension of this loading protocol to cases that until now was not possible to obtain long-time success.

Considering the number of patients included in this study and the missing of RFA data, the findings should be interpreted with care.

Further studies and a greater number of patients are needed to ensure that these new values of insertion torque proposed for immediate loading implants are entirely based on evidence.
